# A monoclonal antibody-based immunoassay for measuring the potency of 2009 pandemic influenza H1N1 vaccines

**DOI:** 10.1111/irv.12272

**Published:** 2014-08-02

**Authors:** Falko Schmeisser, Anupama Vasudevan, Jackeline Soto, Arunima Kumar, Ollie Williams, Jerry P Weir

**Affiliations:** Division of Viral Products, Center for Biologics Evaluation and Research, Food and Drug AdministrationBethesda, MD, USA

**Keywords:** Influenza virus vaccine, monoclonal antibody, potency assay

## Abstract

**Background:**

The potency of inactivated influenza vaccines is determined using a single radial immunodiffusion (SRID) assay. This assay is relatively easy to standardize, it is not technically demanding, and it is capable of measuring the potency of several vaccine strain subtypes in a multivalent vaccine. Nevertheless, alternative methods that retain the major advantages of the SRID, but with a greater dynamic range of measurement and with reduced reagent requirements, are needed.

**Objectives:**

The feasibility of an ELISA-based assay format was explored as an alternative potency assay for inactivated influenza vaccines.

**Methods:**

Several murine monoclonal antibodies (mAbs), specific for the 2009 pandemic H1N1 influenza virus hemagglutinin (HA), were evaluated for their potential to capture and quantify HA antigen. Vaccine samples, obtained from four licensed influenza vaccine manufacturers, included monovalent bulk vaccine, monovalent vaccine, and trivalent vaccine. Traditional SRID potency assays were run in parallel with the mAb–ELISA potency assay using the reference antigen standard appropriate for the vaccine samples being tested.

**Results:**

The results indicated that the ELISA potency assay can quantify HA over a wide range of concentrations, including vaccine at subpotent doses, and the ELISA and SRID potency values correlated well for most vaccine samples. Importantly, the assay was capable of quantifying A/California HA in a trivalent formulation.

**Conclusions:**

This study demonstrates the general feasibility of the mAb approach and strongly suggests that such ELISAs have potential for continued development as an alternative method to assay the potency of inactivated influenza vaccines.

## Introduction

The traditional method used to determine the potency of inactivated influenza vaccines is the single radial immunodiffusion (SRID) assay, essentially as described several decades ago.^1^,^2^ The assay is an agarose gel-based method that measures the diffusion and immunoprecipitation of influenza hemagglutinin (HA) with a strain-specific polyclonal antiserum; the amount of HA antigen present is quantified by comparison with the assigned HA content of a reference antigen standard that is calibrated and distributed by regulatory agencies. The SRID assay is not technically demanding,^3^ and the availability of strain-specific reagents contributes to minimizing interlaboratory variability and assay results, an important consideration as vaccine lots are routinely tested for potency by both manufacturers and regulatory agencies. Importantly, for traditional inactivated influenza vaccines, there is a link between SRID vaccine potency and vaccine immunogenicity^4^–^7^ and a correlation between immunogenicity and clinical benefit.^8^

Nevertheless, there are some disadvantages to the SRID technique that suggest consideration of more modern assay methods. The SRID is not particularly sensitive, it is relatively low throughput,^9^ and the assay format requires large amounts of strain-specific reagents. Several alternative methods for HA quantification have recently been described, including techniques based on HPLC,^10^,^11^ mass spectrometry,^12^–^15^ surface plasmon resonance,^16^ and ELISA^17^,^18^ Several of the techniques that are under evaluation have selective advantages over the SRID and show promise for further development as potential alternative or replacement potency assays. For example, ELISA methods are high throughput, amenable to automation, and miniaturized in comparison with the SRID assay, thus reducing the demand for reagents. The methodology and instrumentation necessary for implementation is widely available, and ELISAs are used as potency assays for other licensed vaccines.

The goal of this study was to assess the feasibility of a monoclonal antibody (mAb)-based ELISA for measuring the potency of inactivated influenza vaccines containing the A(H1N1)pdm09 A/California/7/2009 (A/California) HA. We describe the basic assay set-up and use the mAb-based ELISAs to measure the potency of vaccines produced by four different manufacturers of licensed inactivated influenza vaccine, comparing the results to the potency values produced by the traditional SRID potency. Tested vaccine samples included concentrated monovalent bulk vaccine and final container monovalent vaccine, as well as trivalent vaccines containing A/California HA. In addition, the mAb-based ELISAs were evaluated for their ability to measure the potency of temperature-stressed vaccines. The results indicate that such ELISAs have potential for continued development as an alternative method to assay the potency of inactivated influenza vaccines.

## Materials and methods

### Viruses and monoclonal antibodies

A(H1N1)pdm09 viruses have been described previously.^19^ Reference antigens for influenza vaccine candidates X-179A and X-181 were produced by the Center for Biologics Evaluation and Research in collaboration with licensed vaccine manufacturers. The preparation and initial characterization of eleven monoclonal antibodies to A/California/4/2009 HA have been previously described.^20^

### Potency ELISA

Purified murine monoclonal antibodies were used to coat 96-well immulon-2HB microplates (Dynex Technologies, Chantilly, VA, USA) overnight at 1–2 μg/ml in PBS, followed by washing and blocking with PBS/10% FBS (HyClone, Logan, UT, USA). Reference antigen and vaccine samples were treated with 1% Zwittergent 3-14 for 30 minutes at room temperature as in the SRID assay, but then diluted with PBS to the desired starting concentration (minimum 10-fold dilution). Diluted samples were added to the microplate and serially diluted in PBS and incubated for 2 hours at 37°C. Preliminary studies demonstrated that the Zwittergent pre-treatment step did not affect mAb binding to the antigen. The primary detection antibody was a purified rabbit polyclonal IgG, generated by immunization of rabbits with plasmid DNA vectors expressing A/California HA and boosted with mammalian-derived VLPs^21^ containing the influenza A/California HA. The secondary detection antibody was a goat anti-rabbit IgG conjugated with HRP (KPL, Gaithersburg, MD, USA), and a 1:1 mix of ABTS:H_2_O_2_ (Southern Biotech, Birmingham, AL, USA) was used as enzyme substrate. Plates were read on a VersaMax microplate reader and data generated and analysed with Softmax Pro 5.4.1 (Molecular Devices, Sunnyvale, CA, USA). The HA concentration was determined by parallel line analysis of the four-parameter regression fits of test vaccine samples to that of the reference antigen standard included on each plate. Replicate test samples were included on each plate and replicate plates included in each assay. Assays were repeated a minimum of four times.

### Single radial immunodiffusion

The SRID assay is essentially as described previously^2^,^22^ with minor modifications made more recently.^23^,^24^ Vaccine potency was computed by the parallel line bioassay method using reference and test vaccine dose–response curves (log antigen dilution versus log zone diameter). Average and standard deviation (SD) were calculated from at least four independent tests. For comparisons between SRID results and ELISA potency results, significant differences were analysed using an unpaired, two-tailed Student's *t*-test and were defined as *P* < 0·05 (InStat; GraphPad Prism Software, La Jolla, CA, USA).

## Results

### Characterization of A/California monoclonal antibodies for use as capture antibodies

In a previous study, we described the generation of a panel of murine monoclonal antibodies (mAbs) to the HA of the pandemic influenza H1N1 A/California/04/2009 virus.^20^ We were interested in determining whether these antibodies could be used in an ELISA format to capture and quantify influenza HA as a possible alternative potency assay for inactivated influenza vaccines. In particular, we wanted to determine whether specific antibody characteristics could be defined for the set-up of a successful assay. Five A/California HA-specific mAbs were chosen for evaluation in the ELISA potency.

Table1 summarizes some of the key characteristics of the A/California mAbs that were evaluated in an influenza HA potency ELISA. Previous characterization had shown that all five of the mAbs bound HA1 under reducing conditions in Western blot analysis. Additional studies, which compared the binding of the mAbs to A/California HA at neutral and low pH, also indicated that these mAbs bind the globular head of A/California HA (Figure S1). From the previous study, it was known that mAbs 4F8 and 5C12 had hemagglutination inhibition (HI) activity, were strongly neutralizing in multiple types of neutralization assays and were protective in passive antibody transfer experiments.^20^ Epitope-mapping experiments indicated that these two antibodies competed with each other for the antigenic site Sa on HA, but there was some evidence that suggested that the recognition site for these antibodies might not be identical. The mAbs 4A10 and 3A7 had no measureable HI activity under standard conditions, were weakly neutralizing and were not protective in passive antibody transfer experiments.^20^ However, mAb 4A10 had sufficient HI activity in the presence of complement^25^,^26^ that we were able to select virus escape mutants that localized to the antigenic sites Sb and Ca (Figure S2). The fifth mAb, 1C5, had no measureable HI activity, was not neutralizing, but was partially protective in passive antibody transfer experiments.^20^ In the previous study, 1C5 bound HA much more strongly in a Western blot analysis under non-reducing conditions compared to reducing conditions, suggesting that it might be sensitive to HA conformation.

**Table 1:** Characterization of A/California/4/2009 monoclonal antibodies

mAb	HI*	Neutralization*	Protection*	Epitope	EC_50_ (μg/ml)X181 reference antigen**	EC_50_ (μg/ml)X179A reference antigen**
4F8	Yes	Strong	Yes	HA1 – Sa	0·0089	0·0246
5C12	Yes	Strong	Yes	HA1 – Sa	0·0089	0·0233
4A10	No	Weak	No	HA1 – Sb and Ca	0·0191	0·0648
3A7	No	Weak	No	HA1	0·0195	0·0702
1C5	No	No	Yes	HA1	0·0462	0·0926

HI, hemagglutination inhibition.

* HI, neutralization and protection results for A/California/4/2009 mAbs were reported previously.^20^

** EC_50_, half maximal saturation binding concentration, for each mAb for reference antigens X181 and X179A was determined by sigmoidal dose–response using a four-parameter regression fit (Softmax Pro 5.4.1).

### Set-up of the potency ELISA using A/California mAbs to capture HA

**Figure 1:**
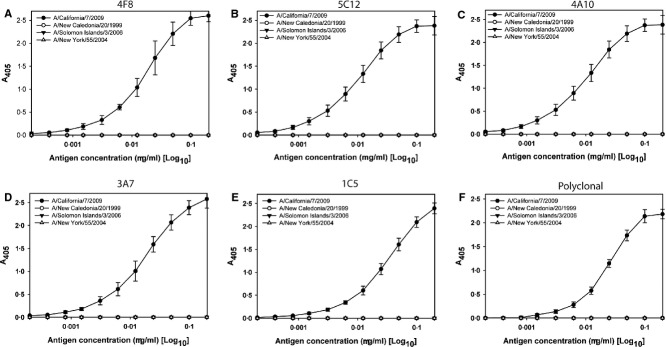
Binding and specificity of mAbs used to capture A/California hemagglutinin (HA). Purified murine mAbs (A–E) and rabbit polyclonal antibody (F) were used to coat ELISA plates at 2 μg/ml prior to binding dilutions of inactivated pandemic H1N1 (A/California), seasonal H1N1 (A/New Caledonia and A/Solomon Islands) and seasonal H3N2 (A/New York) antigen. (A) 4F8 mAb, (B) 5C12 mAb, (C) 4A10 mAb, (D) 3A7 mAb, (E) 1C5 mAb and (F) rabbit polyclonal antibody.

The five A/California HA-specific mAbs were used as capture antibodies for influenza HA in an ELISA format to quantify HA by calculation of the potency of vaccine samples relative to a reference antigen standard with an assigned HA content. Each of the mAbs used in this study were specific for A/California HA, with no apparent binding to HA from seasonal H1N1 or H3N2 viruses (Figure1). The rabbit polyclonal antibody used as the detection antibody in the assay was also highly specific, although it did not appear to capture HA quite as well as some of the mAbs (Figure1F). The relative binding affinities of the five mAbs were calculated from the binding curves using A/California reference antigens X181 and X179A (Table1). HA was bound more strongly by 4F8 and 5C12 than by the other three mAbs (Figure1 and Table1), and each mAb bound X181 reference antigen more strongly than X179A reference antigen.

We obtained vaccine samples from four licensed influenza vaccine manufacturers; these vaccine samples included monovalent bulk vaccine (concentrated), monovalent vaccine and trivalent vaccine, as representative of the various types of vaccine samples that are routinely assayed by SRID in the course of vaccine production. Two different strains of A/California virus were used in the manufacture of these vaccines (two manufacturers each), and consequently, two different reference antigen standards were used for the comparative assays. In all analyses, traditional SRID potency assays were run in parallel with mAb–ELISA potency assays using the reference standard appropriate for the vaccine samples being tested. For presentation of the results, the vaccine samples from the various manufacturers are coded (e.g. Manufacturer 1, 2). ELISA conditions, including mAb coating concentrations, detection antibody concentration, sample treatment and range of reference antigen and test antigen concentrations, were initially optimized using a single reference antigen standard and three monovalent lots of H1N1 A/California vaccine from one manufacturer. These basic conditions were applied to ELISAs using the other types of vaccine samples from other manufacturers, including those using the second reference antigen standard, without further optimization.

### ELISA potency of H1N1 monovalent vaccine bulks

**Figure 2:**
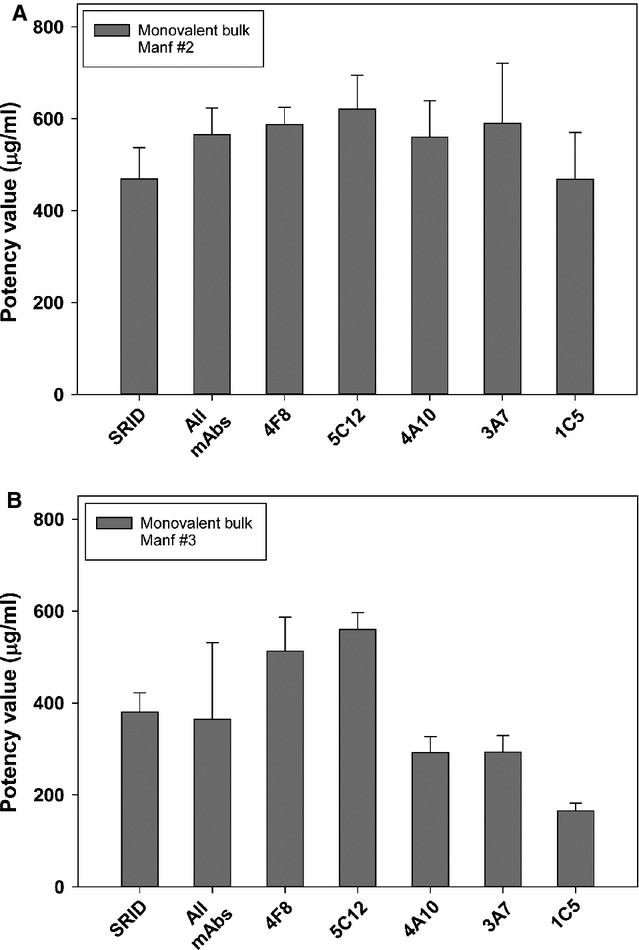
ELISA and single radial immunodiffusion (SRID) potency of A/California monovalent vaccine bulk samples. Potency and standard error of A/California monovalent vaccine bulks (MV) from manufacturer #2 (A) and manufacturer #3 (B) were determined by traditional SRID analysis and by ELISA using five A/California-specific mAbs.

Influenza monovalent bulks, manufactured for each virus strain included in the final vaccine formulation, are generally much more concentrated than the final vaccine. Bulk vaccine lots of A/California vaccine were obtained from two manufacturers and assayed by SRID and ELISA using the five mAbs (Figure2). As expected, the measured HA content for these vaccine bulks was fairly high, ∼400 μg/ml as measured by SRID. ELISA potency values determined using individual mAbs ranged from between 3·5% lower (1C5) to 28% higher (5C12) than the SRID value for the vaccine bulk from manufacturer #2. For the vaccine bulk from manufacturer #3, ELISA potency values determined using 4F8 and 5C12 were 34% and 47% higher, respectively, than the measured SRID value. The ELISA potency values determined using the other three mAbs was lower than the SRID value, ranging from 23% (4A10 and 3A7) to 56% lower. If all five individual mAb–ELISA values were combined, the correlation with the SRID value was greatly improved (17% higher for manufacturer #2 bulk and 4% lower for manufacturer #3) (Figure2 – All mAbs) and was not significantly different.

### ELISA potency of H1N1 monovalent vaccines

**Figure 3:**
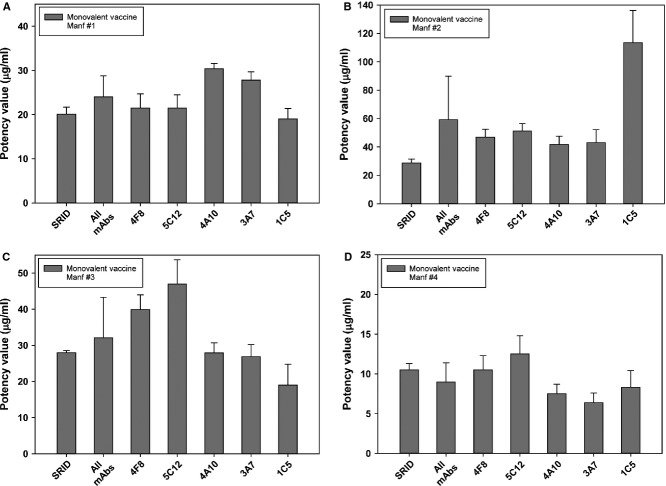
ELISA and single radial immunodiffusion (SRID) potency of A/California monovalent vaccines. Potency and standard error of A/California monovalent vaccines (MVV) from manufacturer #1 (A), and manufacturer #2 (B), manufacturer #3 (C) and manufacturer #4 (D) were determined by traditional SRID analysis and by ELISA using five A/California-specific mAbs.

Monovalent A/California vaccines were obtained from four manufacturers and assayed by SRID and ELISA (Figure3). For monovalent vaccine from manufacturer #1, the ELISA potency values obtained using mAbs 4F8, 5C12 and 1C5 correlated well with the value obtained by SRID (<10% difference). ELISA potency values determined using the mAbs 4A10 and 3A7 were higher (∼40–50%) than the SRID value, but the combined mAb–ELISA value was within 20% of the SRID potency (not statistically different).

ELISA and SRID potency results correlated less well for monovalent vaccine from manufacturer #2. ELISA values obtained with each mAb were higher than the measured SRID value, similar to the pattern observed in the analysis of monovalent bulk vaccine from this manufacturer. However, the ELISA potency value obtained with mAb 1C5 seemed to be an exaggerated outlier, with an ELISA value ∼300% higher than the SRID potency value. Combining this ELISA value with those obtained using the other four mAbs resulted in a combined ELISA potency value much higher than the SRID value, although not statistically different.

For monovalent vaccine from manufacturer #3, the ELISA potency values obtained using mAbs 4A10 and 3A7 correlated extremely well with the value obtained by SRID (<5% difference). ELISA potency values determined using mAbs 4F8 and 5C12 were higher than the SRID value, whereas the 1C5 value was lower than the SRID value. The combined mAb–ELISA potency result was ∼30% higher than the SRID potency value but not significantly different.

The HA content of monovalent vaccine from manufacturer #4 was lower than that from the other manufacturers and was near the limit for accurate quantification by SRID (∼10 μg/ml). Nevertheless, the HA content of this monovalent vaccine was easily measured using the five mAb–ELISAs. The ELISA potency values obtained using mAbs 4F8, 5C12 and 1C5 were close to the value obtained by SRID (0% to ∼20% difference), whereas the ELISA values determined using the other two mAbs were lower than the measured SRID value (∼30–40%). The combined mAb–ELISA value for this monovalent vaccine was within 10% of the SRID potency value.

### ELISA potency of trivalent vaccines containing A/California H1N1

**Figure 4:**
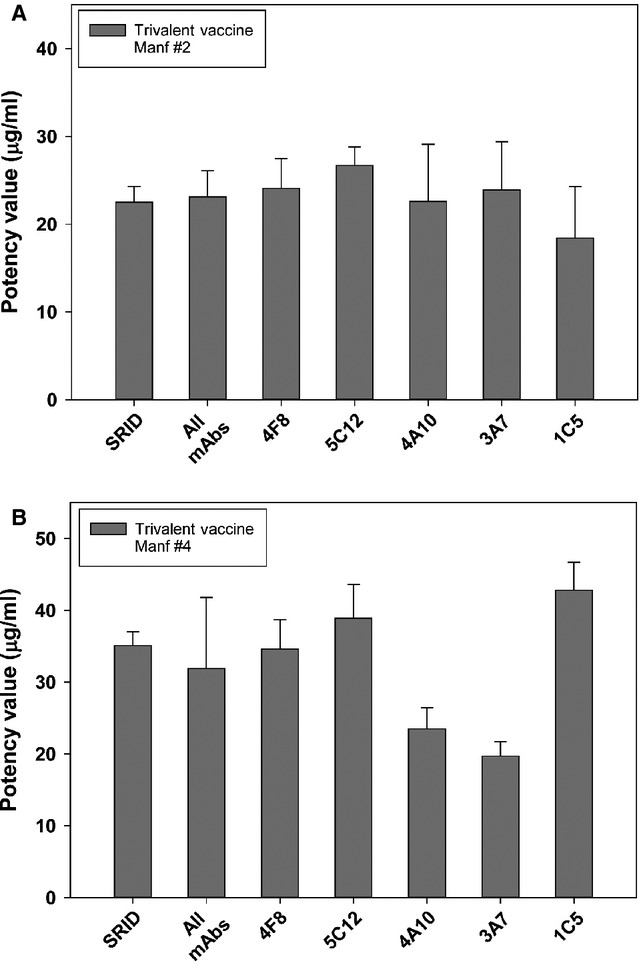
ELISA and single radial immunodiffusion (SRID) potency of trivalent vaccines containing A/California. Potency and standard error of trivalent vaccines containing A/California hemagglutinin (HA) from manufacturer #2 (A) and manufacturer #4 (B) were determined by traditional SRID analysis and by ELISA using five A/California-specific mAbs.

Trivalent influenza vaccine lots containing the A/California strain were obtained from two manufacturers and assayed by SRID and ELISA (Figure4). In addition, trivalent vaccine lots from 2008 to 2009, which contained the seasonal H1N1 strain (A/Brisbane/59/2007) that predated A/California, were obtained from the BEI repository (http://www.beiresources.org). There was no measureable H1N1 potency of this older lot by ELISA using any of the five A/California mAbs (data not shown). For both of the A/California-containing trivalent vaccine lots, however, ELISA potency correlated well with the value obtained by SRID and was not significantly different. For trivalent vaccine from manufacturer #2, the ELISA potency values obtained with each mAb were within 20% of the measured SRID value; mAbs 4F8, 4A10 and 3A7 yielded values within 10% of the SRID value. The combined mAb–ELISA potency was ∼4% higher than the SRID potency value. For trivalent vaccine from manufacturer #4, the combined mAb–ELISA potency was ∼9% lower than the SRID potency value. ELISA potency values obtained with mAbs 4F8, 5C12 and 1C5 yielded values lower than the SRID value, while the other two mAbs yielded higher potency values.

### ELISA potency of temperature-stressed A/California vaccines

**Figure 5:**
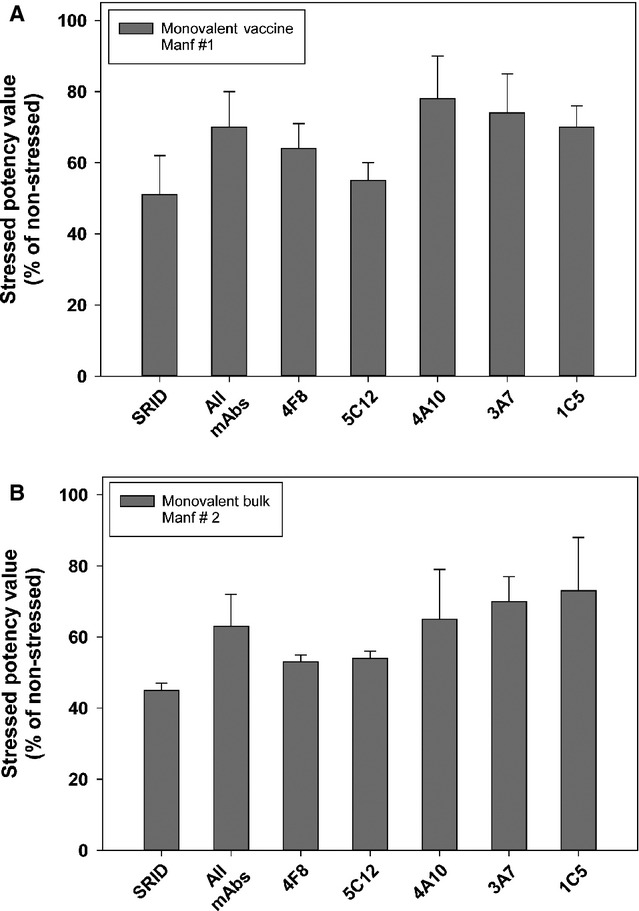
ELISA and single radial immunodiffusion (SRID) potency of A/California vaccine samples subjected to temperature stress. A/California vaccine from manufacturer #1 (A) and A/California vaccine bulk from manufacturer #2 (B) were incubated at 43°C for 1 week before analysis by SRID and ELISA using five A/California-specific mAbs. The percentage drop in potency compared to replicate vaccine samples stored at 4°C is indicated.

We investigated heat treatment to accelerate the decline in vaccine potency with the aim of establishing conditions under which potency was significantly reduced, but not abolished, in a relatively short time frame. An A/California monovalent vaccine lot from one manufacturer and a monovalent vaccine bulk from a second manufacturer were subjected to thermal ageing (43°C – 1 week) and then assayed by SRID and ELISA using the five mAbs (Figure5). Under the thermal ageing conditions, both vaccine lots exhibited a loss in potency of slightly more than 50% by SRID. There was also a decline in potency as measured by ELISA using each of the five mAbs, but the reduction was less than that measured by SRID. For both heat-treated vaccine lots, the ELISA potency values obtained with mAbs 4F8 and 5C12 were lower than the values obtained with the mAbs 4A10, 3A7 and 1C5, and closer to the reduction measured by SRID.

## Discussion

The SRID assay has been the accepted potency assay for inactivated influenza vaccines for over three decades and is used by both vaccine manufacturers and regulatory agencies as a release test for lots of influenza vaccine.^1^,^2^ The assay must be updated routinely as strains included in the vaccine are changed to match the predominant circulating strains of influenza. The production and calibration of new assay reagents, as well as qualification of the updated assay, always present a challenge in the context of the tight timelines inherent in the annual vaccine production cycle.^27^ The relatively low interlaboratory variability of the assay typically results in harmonization of test results obtained by manufacturers and regulatory agencies and also ensures that all inactivated influenza vaccines, which are produced by multiple manufacturers with various different production processes, have similar antigen content and thus clinical benefit.

Nevertheless, the SRID methodology has some weaknesses that suggest the need to explore alternative assays to measure influenza vaccine potency. There are advantages and disadvantages associated with the various methods currently being evaluated, and it is not clear at this time which assay(s) might be a suitable alternative or replacement for the SRID. We have been exploring the development of an ELISA-based assay as a possible alternative potency assay and describe here the set-up of an assay that uses several A/California-specific mAbs as the capture antibodies for HA in the vaccine and in a matched reference antigen standard. Sample preparation in both the ELISA and SRID assay was harmonized, incorporating a Zwittergent detergent treatment step for the standard and vaccine samples. Indeed, in experiments designed to test the effect of the Zwittergent treatment step, we observed much better correlation between ELISA and SRID assay results when standard and vaccine samples were treated with Zwittergent before mAb binding in the ELISA assay (data not shown). We evaluated five mAbs as capture antibodies in the ELISA potency assay. Our results suggest that it may be difficult for an ELISA based on a single mAb to reliably reproduce the results of an SRID assay utilizing a polyclonal antiserum. Further, the results indicate that it is not easy to predict the characteristics of the ELISA mAbs that will yield results closest to SRID results, and additional studies are needed to identify the relevant HA epitopes that are critical for a successful ELISA potency assay. In the absence of such an understanding, antibody properties such as neutralization and HI activity probably should be given a high priority as selection criteria.

Assay results using the two strongly neutralizing antibodies, 4F8 and 5C12, were very similar, regardless of vaccine sample or the vaccine manufacturer. Results obtained using 5C12 differed from those obtained with 4F8 by ∼10%, results that were not unexpected as these two mAbs compete for the same antigenic site on HA. The comparison of two similar, but different, mAbs in this study helps in understanding the variability and reproducibility of the ELISA set-up. The assay results obtained using the mAbs 4A10 and 3A7 were also fairly similar, differing from each other by ∼5%, although there is no data to indicate that these two mAbs recognize the same HA antigenic site. The correlation between the SRID potency results and the ELISA potency results obtained with 4F8/5C12 or 4A10/3A7 varied depending on the type of vaccine sample being analysed and the vaccine manufacturer. Assay results obtained with mAb 1C5 did not follow the pattern of either the 4F8/5C12 or 4A10/3A7 groups of mAbs in terms of correlation with SRID results. Nevertheless, with the unexplained exception of the monovalent vaccine from manufacturer #2, combined ELISA potency results using all mAb assays correlated relatively well with SRID potency values. For monovalent bulks from two manufacturers, monovalent vaccines from three of four manufacturers, and trivalent vaccines from two manufacturers, the combined ELISA potency values and the corresponding SRID values differed by <20%. All three types of vaccine samples were available only from manufacturer #2. Interestingly, whereas there was a relatively poor correlation between the SRID and combined mAb–ELISA values for the monovalent bulk and monovalent vaccine samples from this manufacturer, the combined and individual mAb–ELISA values and the SRID value for the trivalent vaccine sample from this same manufacturer correlated very well.

The ELISA potency assay was able to detect decreased potency in vaccine samples subjected to thermal ageing, although in two independent experiments, a greater decrease was measured by SRID. The drop in potency detected by the 4F8 and 5C12 ELISAs values was closest to the SRID value. While this assay is useful in determining whether specific mAbs can detect a fairly rapid induced potency loss, it is not clear that such an accelerated stability assay is predictive of the ability of the ELISA potency to faithfully reproduce the same drops in potency as would be detected by the SRID for types of stress typically encountered by vaccines. For example, several of the vaccine samples used in this study were well past their expiration dates and had declined in potency over time. The mAb–ELISA potency values determined for these naturally aged vaccine samples correlated very well with concurrent SRID evaluation. Such findings suggest that the correlation between the mAb–ELISA and SRID might be better than predicted by the accelerated thermal ageing experiments, but additional work is needed to define the best methods for assessing the ability of an assay to detect subpotent vaccine.

Other studies have also evaluated the feasibility of an ELISA approach to determining influenza vaccine potency.^17^,^18^,^28^ Indeed, the idea of using a mAb-based ELISA as a potency assay for inactivated influenza vaccines was proposed nearly 30 years ago.^28^ It was noted at the time that the problem of preparing new reagents for every change of vaccine strain might be mitigated using mAbs against relatively persistent antigenic determinants. A much more recent approach used strain-specific mAbs to capture HA antigen followed by detection using the same mAb conjugated to HRP for detection.^17^ The results indicated very good correlation between the mAb–ELISA results and SRID for H1, H3, H5 and influenza B virus strains, as well as the ability to detect subpotent vaccine. This ELISA approach differs somewhat from our approach. Besides the use of a single mAb for both capture and detection, the standards used in the assay of bulk vaccine were preparations of bulk vaccine that were separately quantified by SRID analysis, rather than the reference antigen supplied by regulatory authorities. It seems likely that the incorporation of a Zwittergent detergent treatment step into both the SRID and ELISAs in our study precluded the need for a secondary standard. Another recent ELISA approach to potency assay development utilized synthetic sialic acid receptors to bind HA, followed by detection with strain-specific antibodies.^18^ This ELISA format was also stability-indicating and showed reasonably good correlation with the SRID. Taken together, the results from all of these studies suggest the general feasibility of an ELISA-based approach to influenza vaccine potency determination and that there are likely multiple valid ways to set up such an assay.

In summary, this study describes the set-up and feasibility of a mAb-based ELISA for measuring the potency of inactivated influenza vaccines containing the H1N1pdm09 A/California HA. The results indicate that the assay has the potential to be used for vaccines produced by different manufacturers and suggest that individual vaccine manufacturers might be able to take the reagents described here and optimize the performance of the assay for their own vaccines and vaccine intermediates. The assay can quantify HA over a wide range of concentrations, including vaccine at subpotent doses. Importantly, the assay can quantify A/California HA in a trivalent formulation. Further development of ELISA potency will need to clarify the number and ideal characteristics of mAbs for optimal set-up of the assay and identify the optimal methods for assessing the ability of an assay to detect subpotent vaccine.

## Supporting Information

**Figure S1.** HA1 binding of A/Cal mAbs in a pH-induced conformational change ELISA.

**Figure S2.** Location of HA amino acid changes in A/California escape mutants to mAb 4A10.
