# Supplementary figures and images for: A monoclonal antibody-based immunoassay for measuring the potency of 2009 pandemic influenza H1N1 vaccines
Source: Influenza Other Respir Viruses. 2014 Aug 2;8(5):587–95. doi: 10.1111/irv.12272 (PMC4181825; doi:10.1111/irv.12272)

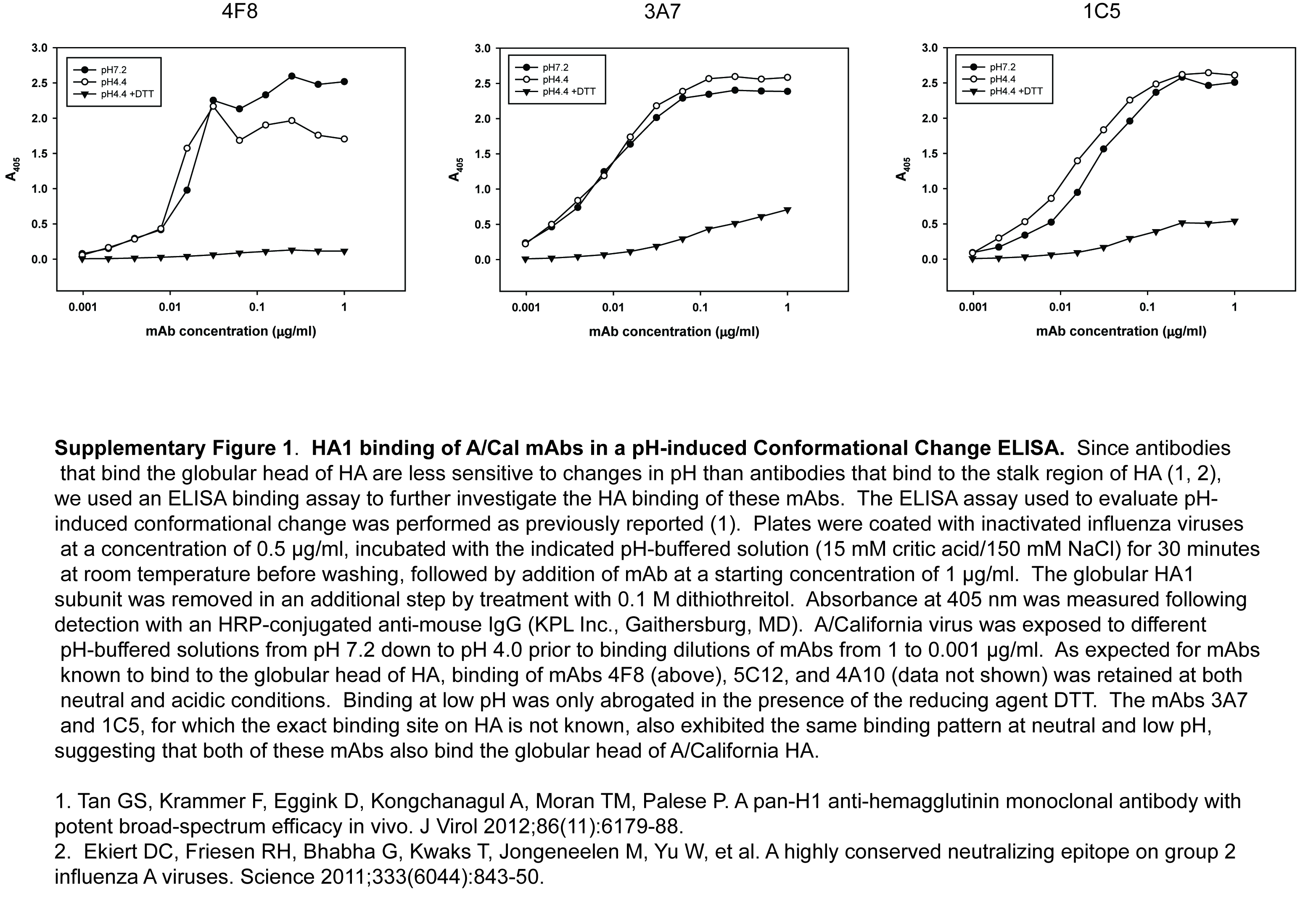

Supplement: Supplementary file 1 — Figure S1. HA1 binding of A/Cal mAbs in a pH-induced conformational change ELISA. [file irv0008-0587-SD1.tif]

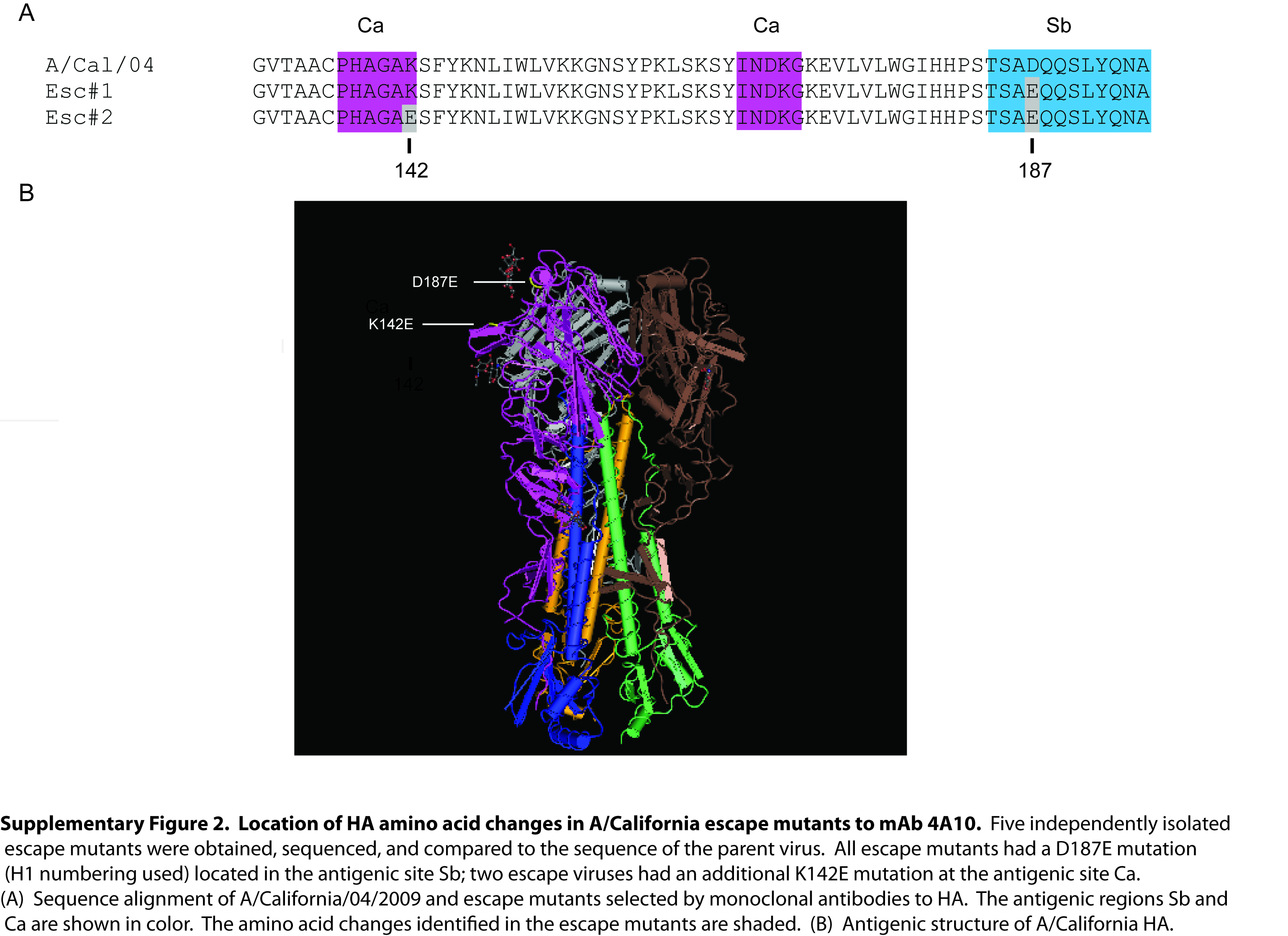

Supplement: Supplementary file 2 — Figure S2. Location of HA amino acid changes in A/California escape mutants to mAb 4A10. [file irv0008-0587-SD2.tif]
